# *Alternaria* Mycotoxins Analysis and Exposure Investigation in Ruminant Feeds

**DOI:** 10.3390/toxins15080495

**Published:** 2023-08-04

**Authors:** Xin Mao, Wanzhao Chen, Huimin Wu, Ying Shao, Ya’ning Zhu, Qingyong Guo, Yanshen Li, Lining Xia

**Affiliations:** 1 Xinjiang Key Laboratory of New Drug Study and Creation for Herbivorous Animals, College of Veterinary Medicine, Xinjiang Agricultural University, Urumqi 830052, China; maoxin103820@126.com (X.M.); cwz752227@163.com (W.C.); m13899623260@163.com (H.W.); 2 College of Life Science, Yantai University, Yantai 264000, China; shaoying@s.ytu.edu.cn (Y.S.); zhuyaning@s.ytu.edu.cn (Y.Z.)

**Keywords:** *Alternaria* mycotoxin, ruminant feeds, LC-MS/MS, analysis, exposure investigation

## Abstract

*Alternaria* mycotoxins are a class of important, agriculture-related hazardous materials, and their contamination in ruminant feeds and products might bring severe toxic effects to animals and even human beings. To control these hazardous compounds, a reliable and sensitive LC-MS/MS (liquid chromatography–tandem mass spectrometry) method was established for simultaneous determination of six target *Alternaria* mycotoxins in ruminant feeds, including ALT (Altenuene), AME (Alternariol Monomethyl Ether), AOH (Alternariol), ATX-Ι (Altertoxins I), TeA (Tenuazonic Acid), and TEN (Tentoxin). This developed analytical method was used for the determination of the presence of these substances in cattle and sheep feeds in Xinjiang Province, China. The results revealed that *Alternaria* mycotoxins are ubiquitously detected in feed samples. Especially, AME, AOH, TeA, and TEN are the most frequently found mycotoxins with a positive rate over 40% and a concentration range of 4~551 µg/kg. The proposed method could be applied for exposure investigation of *Alternaria* mycotoxins in ruminant feeds and for the reduction in the health risk to animals and even consumers.

## 1. Introduction

*Alternaria.* Spp. and mycotoxins are a class of agriculture-related hazardous materials [1]. *Alternaria* is generally observed in cereals, feeds, and agricultural products. These contaminants are toxic secondary metabolites produced by *Alternaria* spp. These toxins usually contaminate fruits, vegetables, grains, and animal feeds, and can enter the body through the food chain [2]. *Alternaria* mycotoxins are reported with five distinct classes based on the major chemical structure [3]. (1) The first is dibenzo-α-pyrones including alternariol (AOH) and alternariol monomethyl ether (AME). (2) The second is perylene quinones including altertoxins I-III (ATX I-III). (3) The third is tetramic acid derivatives including tenuazonic acid (TeA). (4) The fourth is miscellaneous structures including tentoxin (TEN). (5) And the fifth is *A. alternata* f. spp. Lycopersici toxins (AAL-toxins). AOH, AME, and TeA are the most important contaminants that are usually observed in cereals and animal feeds (the most common *Alternaria* toxins are reported in Supplementary Figure S1).

A risk assessment of *Alternaria* mycotoxins was performed by the European Food Safety Authority (EFSA) in 2011. Toxic effects produced by *Alternaria* were reported by numerous experiments both in vivo and in vitro [4], including cytotoxicity, embryotoxicity, genotoxicity, acute toxicity, and lethal toxicity. After exposure of AOH, the cell morphology, cell cycle, and cell activity were reported to be disrupted [5,6,7]. Synergistic effects were also observed after combination exposure of AOH and AME, which could enhance DNA breakage and toxicity [8]. TeA toxicity was observed including diarrhea, muscle tremors and convulsions, dizziness, salivation, and vomiting [9], together with other severe effects, such as tachycardia, massive bleeding from the digestive tract, motor dysfunction, and even death [10,11].

*Alternaria* spp. and mycotoxins commonly contaminate fruits, vegetables, cereals, and feeds all over the world. Among all Alternaria mycotoxins, TeA is the major one with the highest concentrations, followed by AME and AOH. The presence of *Alternaria* mycotoxin in tomato products was demonstrated. From 2017 to 2019 in Italy, a total of 120 tomato samples were gathered in concentrated tomato paste and related products for *Alternaria* mycotoxin contamination investigation [12]. The detection rates of TeA in concentrated tomato paste, tomato paste, tomato puree, and tomato sauce samples were observed as 78.5%, 47.4%, 55.5%, and 76.9%, respectively.

The contamination of *Alternaria* mycotoxins in cereals and feeds might bring heavy economic loss and induce severe toxic effects to animals and human beings [13]. AME, AOH, and TeA contamination were assessed in 872 randomly collected cereal-based foods in China [14]. The detection rates of TeA, AOH, and AME were observed at 47.5%, 7.5%, and 5.7% with mean levels at 18.26 μg/kg (18.00–18.52 μg/kg), 0.23 (0.08–0.37) μg/kg, and 0.66 (0.52–0.30) μg/kg, respectively. Mixed contamination with more than one toxin was observed at 39.9% in the samples analyzed, indicating potential health risks. A three-year survey of the presence and co-occurrence of mycotoxins in a total of 433 cereal-based feed samples in Slovenia was reported [15]. It was revealed that contamination with at least one mycotoxin was noted at 53%. TeA was found in 26% of the grain samples while AOH, TEN, and AME were present in less than 15%. The highest maximum and median concentrations of TeA were 2277 µg/kg and 219 µg/kg in 2016, respectively. The contamination with AME was highest in 2014, whereas the highest maximum and median concentrations were determined in 2015 (1121 µg/kg and 78 µg/kg, respectively). Considering the high frequency and concentration contamination of these hazardous materials, it is necessary to devote more attention to *Alternaria* mycotoxins.

For the exposure investigation of *Alternaria* mycotoxins, various analytical methods were developed including capillary electrophoresis (CE) [16], liquid chromatography–tandem mass spectrometry (LC-MS/MS) [17] and high-resolution mass spectrometry (LC-HRMS) [18], and enzyme-linked immune sorbent assay (ELISA) [19]. However, most of the current analytical methods mainly focused on fruits and related products for hazardous substances’ analysis. There are very few studies in the literature on monitoring these *Alternaria* mycotoxins in cereal-based feeds [20].

Animal husbandry is a pillar industry in Xinjiang Province, China. In 2021, the number of cattle stocks was 9.6 million, with a 12.1% increase compared to 2020. And the number of sheep stock was 48.2 million, with a 5.6% increase compared to 2020 [21]. Considering the toxicity and high-frequency contamination of *Alternaria* mycotoxins, it is important to develop a reliable method for exposure investigation. The aim of this work is to develop a reliable and sensitive LC-MS/MS method for *Alternaria* mycotoxin (ALT, AOH, AME, ATX-Ⅰ, TeA, and TEN) analysis in ruminant animal feeds. In addition, this work also aims to evaluate the animal exposure to mycotoxins through feeds, which will also facilitate the further control of and reduction in possible health risks to animals and consumers.

## 2. Results and Discussion

### 2.1. Optimization of LC-MS/MS Conditions

An individual working solution (1 μg/mL) of each *Alternaria* mycotoxin is infused for full mass and MS/MS spectrum optimization. An integrated positive and negative ESI mode for target analysis is employed in this work. Deprotonated molecular ions [M-H]^−^ of ATX-Ⅰ, TeA, AME, and AOH exhibit a high response in ESI-negative (ESI^−^) mode, while protonated molecular ions [M + H]^+^ of ALT and TEN exhibit a high response in ESI-positive (ESI^+^) mode. One precursor ion (one identification point) is selected according to molecular weight and ionization mode. The three most intense product ions (1.5 identification point for each) are selected for ALT, and the two most intense product ions are selected for AME, AOH, ATX-Ⅰ, TeA, and TEN based on the MS/MS spectrum of the precursor. The optimized MS/MS parameters for qualitative and quantitative analysis (dwell time, cone voltage, and collision energy) for all analytes are presented in Table 1. These developed MS/MS conditions meet the technical criteria of the European Union [22] for target compound identification with at least four identification points.

**Figure 1:**
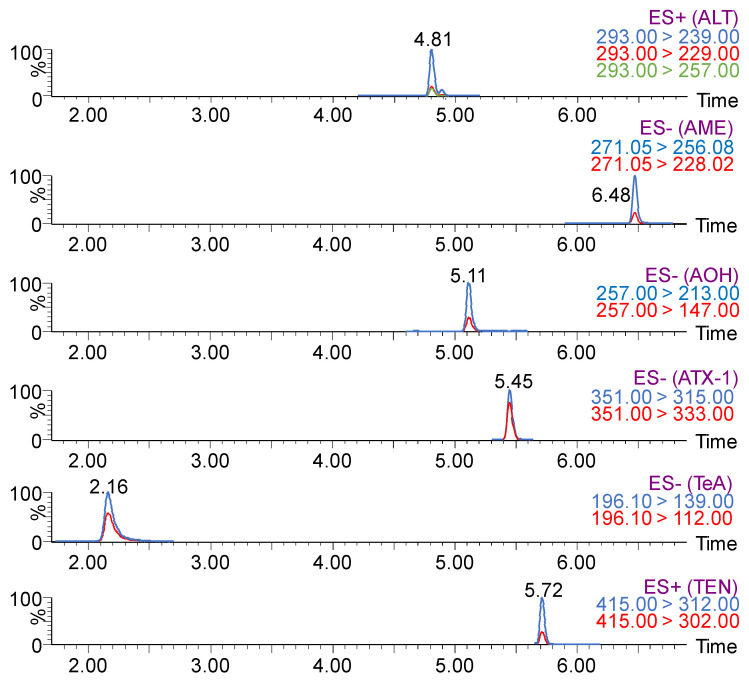
MRM chromatograms of the qualifier ions for the six *Alternaria* mycotoxins in fortified feed samples (prepared by Masslynx 4.2).

Among all targets, the monoacid TeA mycotoxin exhibits a small molecular weight and relatively small retention coefficients in the reversed-phase chromatographic separation system, which could be eluted at an early time in the LC system. In order to avoid potential early eluted matrix interferences with high polarity, the retention time for TeA should be lengthened. The other five substances with low polarity exhibit large retention coefficients to the solid phase. And these compounds should be eluted as quickly as possible. A gradient elution program is selected and optimized for better separation and a sharp response of these targets. In the gradient elution program, the initial mobile phase is confirmed at 85% of solvent A (water containing 1 mM ammonium hydrogen carbonate) and 15% of solvent B (methanol) and maintained for 2.00 min to extend the retention time of TeA. Then, the organic mobile phase of solvent B is increased linearly to 25% until 5.00 min, and the percentage of solvent B continues increasing to 70% until 8.00 min to achieve elution of all mycotoxins. The percentage of solvent B decreases to 15% at 10.00 min and is maintained until 12.00 min for column conditioning. MRM chromatograms with ideal separation and responses of all target mycotoxins are presented in Figure 1.

### 2.2. Optimization of Extraction Procedure

**Figure 2:**
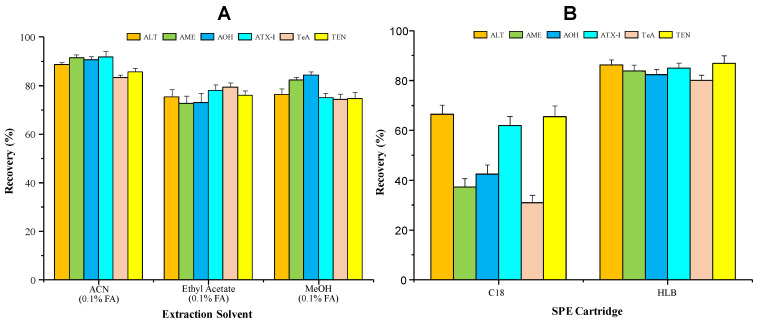
Optimization for extraction with three different solvents (ACN containing 0.1% FA, ethyl acetate containing 0.1% FA, MeOH containing 0.1% FA) (*n* = 3) (**A**) and purification with C18 and HLB cartridges (*n* = 3) (**B**) (prepared by Office Excel 2019).

In order to obtain satisfactory recoveries for all *Alternaria* mycotoxins, the first and critical step is the extraction of targets in the preparation process. In previous reports, the most frequent organic solvents for extraction were MeOH, ACN, and ethyl acetate. ALT, AOH, AME, ATX-Ⅰ, and TEN are soluble in most organic solvents [15,17,23]. TeA is an acidic mycotoxin with pKa at 3.5, and an extraction solvent with a pH value lower than pKa is conductive to promote the distribution of TeA in the organic phase. Therefore, the addition of acid in the extraction solvent is designed to improve recoveries of all targets, especially for the acidic substances (TeA) [24]. And acid types will not be related to the extraction efficiencies according to a previous report [23]. Based on the literature, three different solvents, including ACN containing 0.1% FA, ethyl acetate containing 0.1% FA, and MeOH containing 0.1% FA, are selected for the extraction investigations of target compounds. The optimization of the extraction procedure is processed in triplicates with the concentration at 100 ng/mL for each target (recoveries are shown in Figure 2A). The results show that all three tested solvents could lead to satisfactory recoveries (over 70%), and the highest recoveries are observed when ACN containing 0.1% FA is used (over 80%). And it is selected as the extraction solution for all the substances in this work.

### 2.3. Optimization of Purification Procedure

Co-extracted components might affect the following ionization efficiency in LC-MS/MS analysis [25]. Solid-phase extraction methods for *Alternaria* mycotoxins were described based on recovery experiments in the literature. In this work, Agilent Bond Elut C18 (Agilent Technologies, Inc., Santa Clara, CA, USA) and Waters Oasis HLB (Waters Corporation, Milford, MA, USA) SPE cartridges are chosen for optimization to remove potential co-extracted components with three replicates each (*n* = 3). From the results in Figure 2B, C18 SPE purification could lead to poor results with low recoveries of AME, AOH, and TeA (less than 50%). As for HLB cartridges, satisfactory recoveries (over 80%) for all target compounds could be obtained with 0.1% FA in the condition and washing solvent. The results correspond with those of a previous report where HLB cartridges could be used for solid-phase extraction purification of these substances [23]. In this way, HLB cartridges are used for the *Alternaria* mycotoxin cleanup in this work. Water with 0.1% FA is selected for cartridge condition and sample washing steps during SPE purification.

### 2.4. Method Validation

#### 2.4.1. Selectivity

Target *Alternaria* mycotoxins are identified in spiked feed samples by comparing retention times with the one obtained by commercial standards. Twenty different origin feed samples (10 for cattle and 10 for sheep), which were previously confirmed free of *Alternaria* mycotoxins, are obtained and processed for the evaluation of the selectivity of the developed procedure. The results reveal that chromatograms with RT difference are less than 0.05 min and relative abundance qualitative and quantitative (q/Q) ratios error are less than 15% between calibrators and real samples, which correspond to Guidance SANTE 11312/2021 [26]. MRM chromatograms of the six compounds in fortified feed samples are presented in Figure 1. The results indicate that potential coextracted matrix could be purified after the SPE process with no interferences in the chromatograms.

#### 2.4.2. Linearity

Mixed matrix-matched standard working solutions at six different concentrations are prepared and analyzed based on the optimized LC-MS/MS conditions. Considering the linear range is not from 0, calibration curves are not forced for the point 0:0. Linear regression equations of the eight-point standard curve for all targets are plotted on the basis of peak areas versus different corresponding concentrations, and the correlation coefficient (R^2^) for each is over 0.99 (Table 2). According to the Guidance SANTE 11312/2021 [26], the deviations of the back-calculated concentrations of the calibration standards from the true concentrations using calibration curves in the relevant region are less than ±20%.

Deviation of back calculated concentration (%) = (Cmeasured − Ctrue)/Ctrue × 100%

#### 2.4.3. Sensitivity

Sensitivity is evaluated as described in the literature with LOD and LOQ on the signal-to-noise ratios S/N over 3 and 10, respectively [27,28]. LOD and LOQ ranges of *Alternaria* mycotoxins are 0.147~0.565 µg/kg and 0.488~1.880 µg/kg for cattle feeds, while they are 0.167~0.548 µg/kg and 0.556~1.825 µg/kg for sheep feeds (Table 2), respectively. The sensitivity of this developed method is higher than those in previous reports in fruits and juices [29], sweet pepper [30], cereals [31], and animal feeds [15].

#### 2.4.4. Accuracy and Precision

Accuracy and precision are assessed based on recovery experiments of spiked feed samples at 5, 10, and 20 μg/kg. RSDr (relative standard deviation in repeatability conditions) (*n* = 6) and RSDwr (relative standard deviation in within-lab reproducibility conditions) (*n* = 3) precisions are evaluated with six replicates of spiked feed samples on one day and three continuous days, respectively (results are presented in Table 3). From the table, mean recoveries are in the range of 78~100% for cattle feeds with RSDr and RSDwr less than 10% and 7%, respectively. As for sheep feeds, mean recoveries are in the range of 78~99% with RSDr and RSDwr less than 12% and 9%, respectively. According to Guidance SANTE 11312/2021, the results indicate an acceptable accuracy and precision in this developed procedure [26].

### 2.5. Exposure of Alternaria Mycotoxins in Ruminant Feeds

To estimate the exposure of *Alternaria* mycotoxins in ruminant feeds, a total of 40 feed samples (20 cattle feeds and 20 sheep feeds) are collected from Xinjiang Province, China. All samples are treated and analyzed according to this developed protocol for target analysis. Samples containing these contaminants with higher concentrations than the linear range could be diluted with processed corresponding blank sample solution before quantification. And the results are presented in Table 4 and Table S1. From the table, it can be concluded that AME, AOH, and TeA are the three major contaminants in ruminant feeds, while ALT is not detected in all samples. In tested cattle feeds, 12, 15, 12, and 10 samples are detected positive for AME, AOH, TeA, and TEN, respectively, while in all tested sheep feeds, the number of positive samples are 15, 13, 10, and 8 for AME, AOH, TeA, and TEN, respectively. It is also observed that all 10 cattle feed samples with TEN positive are detected to be co-occurring of AME, AOH, and TeA, while only six sheep feed samples are detected to be co-occurring with AME, AOH, TeA, and TEN. In addition, ATX-I is also detected in feed samples with a positive rate at 10% for cattle feeds and 15% for sheep feeds.

Interestingly, sheep feeds seem to be slightly more contaminated with higher concentrations than cattle feeds. In sheep feeds, TeA concentrations are higher than the others with the highest concentrations at 551 µg/kg, which corresponds with a previous report on TeA in wheat floor in China [32]. The lower concentration of *Alternaria* mycotoxins might be due to the 1~5% salt as one of the major compositions in cattle feeds. AME, AOH, TeA, and TEN are the major contaminated toxins, while ALT exhibits a rare contamination rate. This is in line with the literature on fruits, tomatoes, cereals, and related products contaminated by a considerable amount of *Alternaria* mycotoxins [17,33,34].

## 3. Conclusions

In this work, a sensitive and reliable LC-MS/MS method is established for simultaneous determination of six *Alternaria* mycotoxins, including ALT, AME, AOH, ATX-Ι, TeA, and TEN in ruminant feeds. After extensive optimization of extraction and purification approaches, satisfactory recovery, favorable sensitivity, and low limits of detection for all targets could be achieved. This developed analytical method is successfully applied for the determination of the exposure of these substances in cattle and sheep feeds in Xinjiang Province, China. The results reveal that *Alternaria* mycotoxins are ubiquitously detected in feed samples. Especially, AME, AOH, TeA, and TEN are the major contaminated hazardous compounds in feed samples. The proposed method could be applied for the continuous monitoring of *Alternaria* mycotoxins in feeds and the reduction in the health risk to animals and even consumers.

## 4. Materials and Methods

### 4.1. Chemicals and Reagents

**Figure 3:**
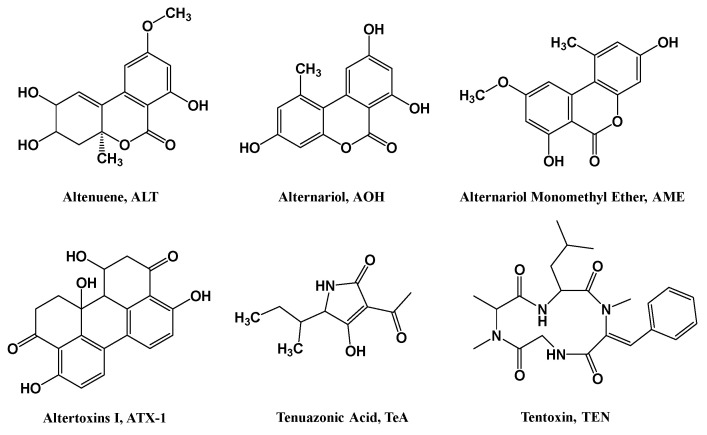
Chemical structures of *Alternaria* mycotoxins (prepared by ChemDraw 18.0).

Commercial standards of *Alternaria* mycotoxins, including Altenuene (ALT), Alternariol Monomethyl Ether (AME), Alternariol (AOH), Altertoxins I (ATX-Ⅰ), Tenuazonic Acid (TeA), and Tentoxin (TEN) were obtained from Pribolab Pte. Ltd. (Immunos, Singapore). (Chemical structures are presented in Figure 3). Acetonitrile, methanol, ethyl acetate, and formic acid (HPLC grade) were available from Thermo Fisher Scientific Inc. (Mullica Hill, NJ, USA). Other reagents (analytical grade) applied in this work were obtained from Sinopharm Chemical Reagent Co., Ltd. (Beijing, China).

### 4.2. Apparatus

An HQ-60 vortex mixer was obtained from North TZ-Biotech Development Co. Ltd. (Beijing, China). Milli-Q system (Millipore, Burlington, MA, USA) was obtained for the deionized water preparation. Nitrogen evaporation was carried out using a product from Organomation Associates Inc. (Berlin, MA, USA). Syringe filters (0.2 μm) were obtained from Pall Corp. (Ann Arbor, MI, USA). A refrigerated centrifuge (3 k 15) was obtained from Sigma Laborzentrifugen GmbH (Osterode am Harz, Germany). OASIS HLB cartridges (60 mg, 3 cc) were obtained from Waters Corporation (Milford, MA, USA).

### 4.3. Standard Solution Preparation

Each *Alternaria* mycotoxin stock solution (1 mg/mL) was prepared by dissolving 1 mg of each standard in 1 mL of acetonitrile. Individual working solution (100 μg/mL) was prepared by diluting 100 μL of each *Alternaria* mycotoxin with acetonitrile to a final volume of 1 mL. Mixed working solution (100 μg/mL of each) was prepared by adding 100 μL of each *Alternaria* mycotoxin stock solution (1 mg/mL) to a new vial and diluted with acetonitrile to a final volume of 1 mL. The dilution and preparation of these prepared standard solutions were verified by calculating the concentration to ensure the reliability. These solutions were kept at −20 °C. The reconstitution solution for the dissolution of targets before LC-MS/MS was methanol/water (75/25, *v*/*v*) containing 1% formic acid.

### 4.4. Sample Preparation

The preparation of feed samples for *Alternaria* mycotoxins analysis was processed according to a previous publication [35] with some modification. Feed samples (500 g) were ground for 2 min by using an Osterizer and filtered through 50 mesh sieves. Then, 2.00 ± 0.02 g of feed samples was weighed into a 50 mL screw cap test tube. Feed samples were fortified with six standards for method validation analysis with final concentrations at 5, 10, and 20 μg/kg by adding 10, 20, and 40 μL of mixed working solution, respectively. One was settled as the negative control without any spiking. After fortification, each sample was placed in a dark place for 30 min for solvent evaporation and incubation to a simulated natural contamination. Acetonitrile containing 0.1% FA was utilized as the extract solvent, and the extraction procedure for targets was performed by adding 20 mL of extract solvent and vortexing for 3 min. After centrifugation at 12,000× *g* for 10 min at 4 °C, the supernatant was collected and evaporated under a gentle steam of nitrogen at 60 °C. The residue was re-dissolved by using 3 mL of deionized water for further SPE purification with OASIS HLB cartridges. Each cartridge was conditioned with 3 mL of acetonitrile containing 0.1% FA and 3 mL of water containing 0.1% FA in turn. Then, each sample was loaded on conditioned cartridge by gravity. After washing with 3 mL of acetonitrile/water (1:9, *v*/*v*) containing 1% formic acid, each sample was eluted with 5 mL of acetonitrile. The elute was dried by using nitrogen evaporation at 60 °C and the residue was reconstituted with 500 μL of methanol/water (75/25, *v*/*v*) containing 1% formic acid. Each sample was filtered through 0.2 μm syringe filters before LC-MS/MS analysis.

### 4.5. LC-MS/MS Parameters

An ultra-high-performance liquid chromatography system (UPLC) (Waters, Millford, MA, USA) equipped with a BEH C8 column (2.1 mm × 50 mm, 1.7 μm) was utilized for target separation. Water containing 1 mM ammonium hydrogen carbonate was used as solvent A and methanol was used as solvent B. To obtain satisfactory separation results of all targets, a gradient elution program was used and the column oven was maintained at 30 °C. The gradient elution program was as follows: 0–2.0 min, 15% B; 2.0–5.0 min, 15–25% B; 5.0–8.0 min, 25–70% B; 8.0–10.0 min, 70–15% B; 10.0–12.0 min 15% B.

Waters Xevo TQXS (Milford, MA, USA) triple-quadrupole mass spectrometer was coupled to UPLC for mass spectrum analysis by using multiple reaction monitoring (MRM) mode. The mass spectrum conditions for MRM transitions and collision energies were optimized for each target on the basis of the MS response (Supplementary Materials). Technically, each sample was run in three replicates and the average value was used for further analysis.

### 4.6. Method Validation

In this work, method specificity, sensitivity, linearity, accuracy, and precision were validated in spiked feed samples. Specificity was assessed by comparing with the blank control with spiked feed samples to ensure there were no interfering peaks present at the retention time of each target. Sensitivity was evaluated by limits of detection (LODs) and limits of quantification (LOQs). The LOD and LOQ of each analyte were evaluated based on signal-to-noise ratio S/N. The LOD was determined by S/N ≥ 3, while LOQ was S/N ≥ 10. Linearity was evaluated with matrix-spiked calibration curves at concentrations of 2.0, 5.0, 10.0, 20.0, 50.0, 100.0, 200.0, and 500.0 ng/mL. Accuracy and precision were evaluated by analyzing QC samples at three different spiked levels (5, 10, and 20 μg/kg). QC samples of each level were processed with six replicates of spiked feed samples on one day and three continuous days as biological replicates. The concentration of each sample was calculated based on the matrix-spiked calibration curve. Accuracy was evaluated as recoveries of spiked samples, which was evaluated with the following equation. Precision is expressed as the RSDr (relative standard deviation in repeatability conditions) (*n* = 6) and RSDwr (relative standard deviation in within-lab reproducibility conditions) (*n* = 3).

$$\mathrm{Recovery}=\frac{\mathrm{mean}\;\mathrm{calculated}\;\mathrm{concentration}}{\mathrm{nominal}\;\mathrm{concentration}}\times 100\%$$

### 4.7. Exposure Investigation of Alternaria Mycotoxins in Commercial Ruminant Feeds

In order to investigate *Alternaria* mycotoxin exposure levels, 40 feed samples (20 cattle feeds and 20 sheep feeds) were obtained from local farms and Taobao Alibaba online malls located in Xinjiang Province. Feed samples were processed and analyzed with the developed and validated LC-MS/MS protocol. Concentrations of target hazardous substances in feed samples were determined on the basis of the matrix spiked calibration curve.

## Figures and Tables

**Table 1:** The optimized MS/MS parameters for qualitative and quantitative analysis (dwell time, cone voltage, and collision energy) for all analytes are presented in Table 1.

Mycotoxin	Ionization Mode	Precursor (*m*/*z*)	Product (*m*/*z*)	Dwell (s)	Cone Voltage (V)	Collision Energy (eV)
ALT	ESI^+^	293.00	229.00	0.016	33.0	20.0
			239.00 *			25.0
			257.00			15.0
AME	ESI^−^	271.05	228.02	0.039	30	30.0
			256.08 *			22.0
AOH	ESI^−^	257.00	147.00	0.022	64.0	32.0
			213.00 *			24.0
ATX-Ⅰ	ESI^−^	351.00	315.00 *	0.135	20.0	15.0
			333.00			10.0
TeA	ESI^−^	196.10	112.00	0.080	52.0	24.0
			139.00 *			18.0
TEN	ESI^+^	415.00	302.00	0.039	40.0	18.0
			312.00 *			18.0

* Stands for quantifier ion.

**Table 2:** Linearity, LOD, and LOQ results of *Alternaria* mycotoxins in different ruminant feeds.

Matrix	Mycotoxin	Linear Regression	Linear Range (ng/mL)	*R* ^2^	LOD (µg/kg)	LOQ (µg/kg)
Cattle Feed	ALT	*y* = 1267 *x* + 7908	2.0–500.0	0.9963	0.316	1.052
	AME	*y* = 2096 *x* − 3561	2.0–500.0	0.9988	0.356	1.184
	AOH	*y* = 1954 *x* − 7781	2.0–500.0	0.9994	0.320	1.064
	ATX-Ι	*y* = 2986 *x* + 5319	2.0–500.0	0.9969	0.193	0.641
	TeA	*y* = 2148 *x* + 3501	2.0–500.0	0.9949	0.565	1.880
	TEN	*y* = 1124 *x* + 5482	2.0–500.0	0.9983	0.147	0.488
Sheep Feed	ALT	*y* = 804 *x* + 1528	2.0–500.0	0.9958	0.272	0.906
	AME	*y* = 998 *x* + 1506	2.0–500.0	0.9939	0.452	1.505
	AOH	*y* = 1051 *x* + 1122	2.0–500.0	0.9967	0.393	1.309
	ATX-Ι	*y* = 1280 *x* + 1597	2.0–500.0	0.9979	0.168	0.560
	TeA	*y* = 797 *x* + 2510	2.0–500.0	0.9938	0.548	1.825
	TEN	*y* = 1148 *x* + 2548	2.0–500.0	0.9927	0.167	0.556

**Table 3:** Accuracy and precision results in fortified ruminant feeds.

Mycotoxin	Matrix	Fortified Level (µg/kg)	Mean Recovery (%)	RSDr % (*n* = 6)	RSDwr % (*n* = 3)	Matrix	Fortified Level (µg/kg)	Mean Recovery (%)	RSDr % (*n* = 6)	RSDwr % (*n* = 3)
ALT	Cattle Feed	5	91	8	4	Sheep Feed	5	86	9	6
		10	94	5	2		10	91	12	9
		20	100	8	4		20	86	7	3
AME		5	96	9	5		5	92	7	3
		10	85	10	7		10	81	12	9
		20	82	9	6		20	94	9	5
AOH		5	85	6	2		5	83	9	5
		10	89	10	7		10	96	9	6
		20	85	8	5		20	88	10	7
ATX-Ι		5	93	6	3		5	76	10	5
		10	88	5	1		10	86	8	4
		20	95	7	3		20	78	7	4
TeA		5	78	9	5		5	85	9	5
		10	84	10	6		10	85	8	5
		20	91	6	2		20	91	9	5
TEN		5	82	8	4		5	80	9	5
		10	91	7	4		10	99	7	3
		20	96	7	4		20	89	10	7

**Table 4:** Occurrence and levels of *Alternaria* mycotoxins in ruminant feeds.

Feeds	Mycotoxin	Positive	Concentration (µg/kg)	Median (µg/kg)
Cattle	ALT	0	ND	ND
	AME	60%	39~238	180
	AOH	75%	22~196	156
	ATX-Ι	10%	4~9	6
	TeA	60%	56~295	189
	TEN	50%	4~39	22
Sheep	ALT	0	ND	ND
	AME	75%	62~481	396
	AOH	65%	66~386	255
	ATX-Ι	15%	6~15	8
	TeA	50%	87~551	450
	TEN	40%	8~142	82

ND: not detected. Lower than LOD of 0.316 µg/kg in cattle feeds and 0.272 µg/kg in sheep feeds for ALT.

## Data Availability

Not applicable.

## Supplementary Materials

The following supporting information can be downloaded at: https://www.mdpi.com/article/10.3390/toxins15080495/s1, Figure S1. Chemical structures of the most common *Alternaria* toxins (Prepared by Chem Draw 18.0). Figure S2. Chromatograms of each analyte at the lowest calibration curve level (2 ng/mL) (Prepared by Masslynx 4.2). Table S1. Occurrence and levels of *Alternaria* toxins in ruminant feeds (*n* = 40). Table S2. Most optimized mass spectrum parameters for *Alternaria* mycotoxins.
