# Supplementary material for: Alternaria Mycotoxins Analysis and Exposure Investigation in Ruminant Feeds
Source: Toxins (Basel). 2023 Aug 4;15(8):495. doi: 10.3390/toxins15080495 (PMC10467096; doi:10.3390/toxins15080495)
Supplement: Supplementary file 1 [file toxins-15-00495-s001.zip › toxins-2478562-supplementary.pdf]

toxins

*Article*

# *Alternaria* Mycotoxins Analysis and Exposure Investigation in Ruminant Feeds

**Xin Mao [1], Wanzhao Chen [1], Huimin Wu [1], Ying Shao [2], Ya'ning Zhu [2], Qingyong Guo [1,*], Yanshen Li [2,*] and Lining Xia [1,*]**

[1] Xinjiang Key Laboratory of New Drug Study and Creation for Herbivorous Animals, College of Veterinary Medicine, Xinjiang Agricultural University, Urumqi 830052, China; maoxin103820@126.com (X.M.); cwz752227@163.com (W.C.); m13899623260@163.com (H.W.)

[2] College of Life Science, Yantai University, Yantai 264000, China; shaoying@s.ytu.edu.cn (Y.S.); zhuyaning@s.ytu.edu.cn (Y.Z.)

* Correspondence: dygqy@edu.xjau.cn (Q.G.); liyanshen@ytu.edu.cn (Y.L.); xln@edu.xjau.cn (L.X.); Tel.: +86-991-8762704 (Q.G.); +86-535-6902638 (Y.L.); +86-991-8763012 (L.X.)

*LC-MS/MS parameters*

Optimized mass spectrum parameters were settled as follows: Ion Spray Voltage, 3.0 KV, Cone Gas Flow, 50 L/h, Desolvation Gas Flow, 700 L/h, Source Temperature, 150 °C, and Desolvation Temperature, 500 °C. Argon was used as collision gas and the pressure was at 0.8 mbar.

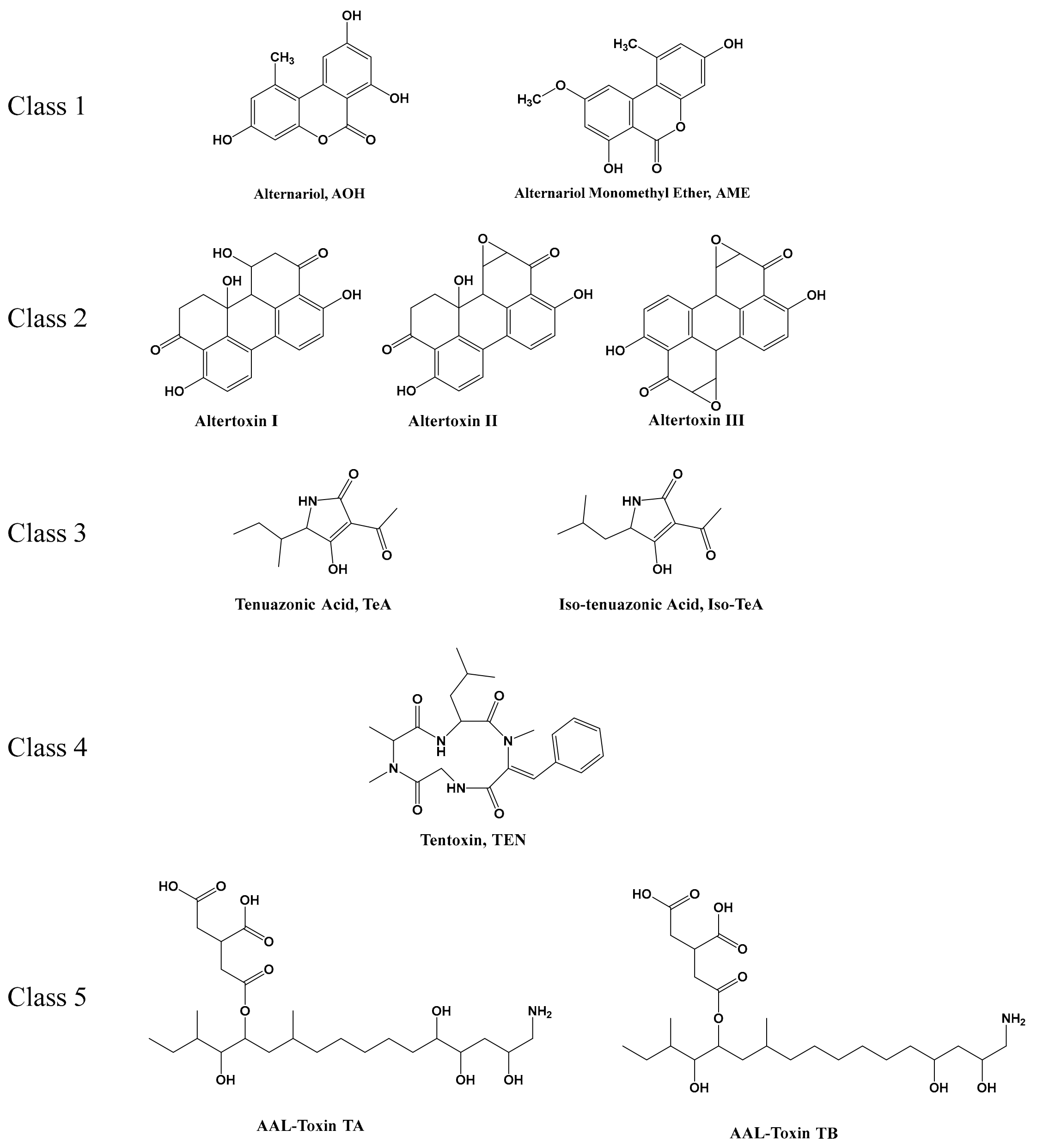


**Figure S1.** Chemical structures of the most common *Alternaria* toxins (Prepared by Chem Draw 18.0).

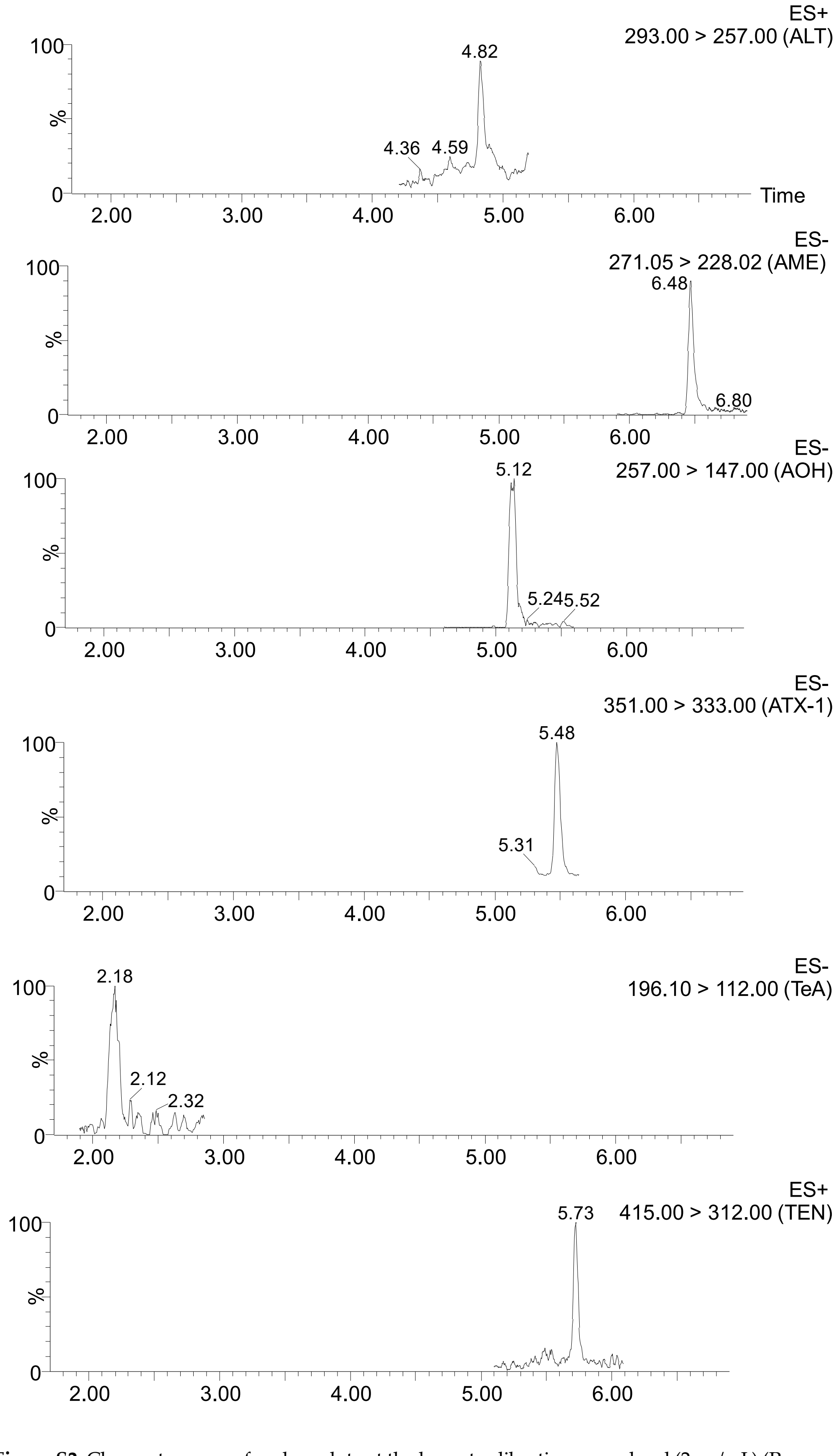


**Figure S2.** Chromatograms of each analyte at the lowest calibration curve level (2 ng/mL) (Prepared by Masslynx 4.2).

**Table S1.** Occurrence and levels of *Alternaria* toxins in ruminant feeds ($n = 40$).

| Type of Feed | Sample No. | AME (μg/kg) | AOH (μg/kg) | ATX-I (μg/kg) | TeA (μg/kg) | TEN (μg/kg) |
|---|---|---|---|---|---|---|
| Cattle Feeds | 1 | 39 | 22 | 4 | 56 | 4 |
| | 2 | 238 | 196 | 9 | 295 | 39 |
| | 3 | ND | ND | ND | ND | ND |
| | 4 | 196 | 178 | ND | 276 | 10 |
| | 5 | 208 | 189 | ND | 206 | 22 |
| | 6 | 164 | 149 | ND | 150 | 6 |
| | 7 | ND | ND | ND | ND | ND |
| | 8 | 155 | 141 | ND | 195 | 18 |
| | 9 | 85 | 133 | ND | 165 | 25 |
| | 10 | ND | ND | ND | ND | ND |
| | 11 | ND | ND | ND | ND | ND |
| | 12 | ND | ND | ND | ND | ND |
| | 13 | 92 | 84 | ND | 175 | 8 |
| | 14 | 219 | 199 | ND | 177 | 8 |
| | 15 | 209 | 190 | ND | 182 | 10 |
| | 16 | 203 | 185 | ND | 233 | ND |
| | 17 | 109 | 99 | ND | 265 | ND |
| | 18 | ND | 162 | ND | ND | ND |
| | 19 | ND | 151 | ND | ND | ND |
| | 20 | ND | 156 | ND | ND | ND |
| Sheep Feed | 21 | 58 | 66 | 6 | 87 | 8 |
| | 22 | 62 | 87 | 15 | 551 | 142 |
| | 23 | 82 | 97 | 8 | 161 | 65 |
| | 24 | 98 | 99 | ND | 182 | 48 |
| | 25 | 350 | 99 | ND | 199 | 106 |
| | 26 | ND | ND | ND | ND | ND |
| | 27 | ND | ND | ND | 464 | ND |
| | 28 | 384 | 255 | ND | 489 | 122 |
| | 29 | ND | ND | ND | ND | ND |
| | 30 | 396 | 258 | ND | 502 | 86 |
| | 31 | 400 | 315 | ND | 496 | ND |
| | 32 | 415 | 331 | ND | 436 | ND |
| | 33 | 443 | 353 | ND | ND | ND |
| | 34 | 466 | 360 | ND | ND | ND |
| | 35 | 470 | 386 | ND | ND | ND |
| | 36 | 479 | ND | ND | ND | ND |
| | 37 | 481 | ND | ND | ND | ND |
| | 38 | ND | ND | ND | ND | ND |
| | 39 | ND | ND | ND | ND | ND |
| | 40 | 367 | 223 | ND | ND | 78 |

Each sample was run in three replicates.

Sample No. (1-20) stands for twenty different batches of cattle feed, while sample No. (21-40) stands for twenty different batches of sheep feed.

ND: not detected. Lower than LOD.

**Table S2.** Most optimized mass spectrum parameters for *Alternaria* mycotoxins.

| Parameter | Value |
|---|---|
| Ion Spray Voltage | 3.0 KV |
| Cone Gas Flow | 50 L/h |
| Desolvation Gas Flow | 700 L/h |
| Source Temperature | 150 °C |
| Desolvation Temperature | 500 °C |
| Collision Gas | Argon |
| Pressure | 0.8 mbar |
